# Advances in the study of CDC42 in the female reproductive system

**DOI:** 10.1111/jcmm.17088

**Published:** 2021-12-03

**Authors:** Qiaojuan Mei, Huiying Li, Yu Liu, Xiaofei Wang, Wenpei Xiang

**Affiliations:** ^1^ Institute of Reproductive Health and Center for Reproductive Medicine Tongji Medical College Huazhong University of Science and Technology Wuhan China

**Keywords:** CDC42, meiosis, oocyte, reproductive system, Rho‐GTPase

## Abstract

CDC42 is a member of the Rho‐GTPase family and is involved in a variety of cellular functions including regulation of cell cycle progression, constitution of the actin backbone and membrane transport. In particular, CDC42 plays a key role in the establishment of polarity in female vertebrate oocytes, and essential to this major regulatory role is its local occupation of specific regions of the cell to ensure that the contractile ring is assembled at the right time and place to ensure proper gametogenesis. The multifactor controlled ‘inactivation‐activation’ process of CDC42 also allows it to play an important role in the multilevel signalling network, and the synergistic regulation of multiple genes ensures maximum precision during gametogenesis. The purpose of this paper is to review the role of CDC42 in the control of gametogenesis and to explore its related mechanisms, with the aim of further understanding the great research potential of CDC42 in female vertebrate germ cells and its future clinical translation.

## 1 INTRODUCTION

Cell division cycle 42 (CDC42) is a member of the Rho‐GTPase family. It is 191 amino acids in length, located on chromosome 1p36.12, and it contains a molecular weight of 21.33 kDa.^1^ CDC42 was first identified in budding yeast in 1997, and it plays an integral role in the regulation of cell motility, cell proliferation, cytoskeleton and in particular, microfilament‐dependent cell polarity.^2^, ^3^, ^4^, ^5^ CDC42 is highly homologous and conserved in human and yeast, and can be hypothesized to play a fundamental role in cell biology.^6^ CDC42 also plays a central role in the establishment of cell polarity.^7^ The first evidence of the involvement of CDC42 in the establishment of cell polarity came from a study by Adams et al. in yeast,^8^ which showed that a novel effector domain of CDC42 is involved in triggering the yeast G2/M transition and can couple the cell cycle to play a role in cytoskeletal rearrangements.^9^ Later, in Drosophila oocytes, it was found that Drosophila maternal effector proteins interact with both actin through the WH2 structure and CDC42, thereby localizing GTPase to the actin cytoskeleton.^10^ It is noteworthy that the multifactor‐controlled ‘inactivation‐activation’ process of CDC42 also makes it play an important role in the combined regulation of multiple genes and signalling pathway network, and the synergistic effect of multiple genes makes the process of female gametogenesis more precise. Thus, the role of CDC42 in female vertebrate gametogenesis has been gradually unveiled. In this context, the aim of this paper is to review the role played by CDC42 in controlling female gametogenesis and to summarize the related mechanisms. We will describe how CDC42 regulates female gametogenesis (including four chapters on follicular reserve and primordial follicle activation, meiosis, fertilization and embryo formation); similarly, we will briefly describe the role of CDC42 in the granulosa cells, which are inseparable from the oocyte, in order to understand the role of CDC42 in female reproductive development and to provide further insight into the role of CDC42 in female reproductive development.

## 2 METHODS

**FIGURE 1:**
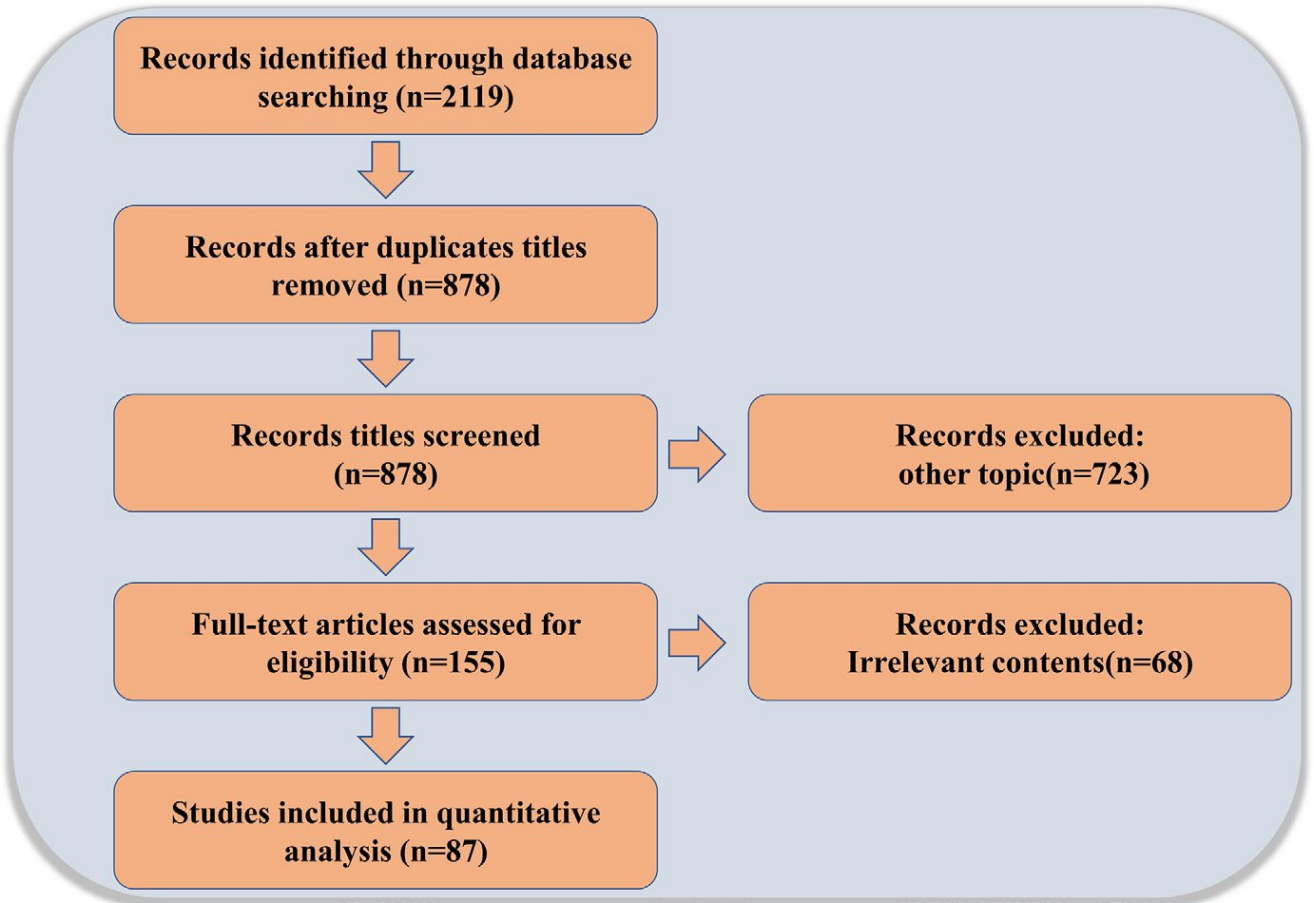
Schematic of study selection

For this review, we conducted a systematic online literature search of PubMed and Web of Science databases using a total of three strategies including literature search, study selection and summary of results, and searched all published articles since the creation of the databases up to 2021. We used the following queries: (‘cdc42 GTP‐Binding Protein’ or ‘cdc42 protein’ or ‘Cell division cycle 42’ or ‘CDC42’) and (‘primordial follicle formation’ or ‘follicle growth’ or ‘ovulation’ or ‘fertilization’ or ‘embryo’). Both animal and human studies were considered appropriate for this review. In addition, all relevant studies were identified and included. Any duplicate articles were excluded. After screening the titles and/or abstracts, articles were excluded if they were found to be irrelevant to the study. A total of 2119 records were retrieved from both databases. After excluding duplicate titles and other subject articles, the full text of 146 articles was reviewed and 87 were considered relevant and included in this review (Figure 1).

## 3 OVARIAN RESERVE AND FOLLICULOGENESIS

**FIGURE 2:**
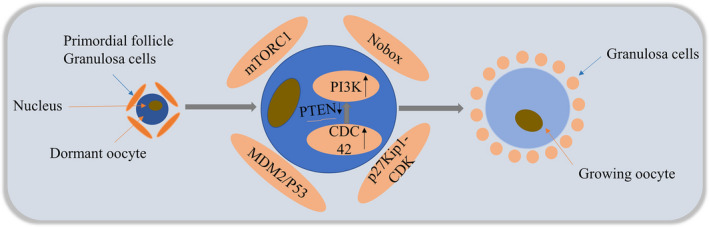
Current models of primordial follicle activation in the mammalian ovary. In the adult mammalian ovary, only a small fraction of dormant primordial follicles are awakened to further developmental stages. Several signalling pathways exist to control primordial follicle activation, such as the mTORC1 signalling system, the p27Kip1‐CDK signalling pathway, MDM2/P53 and Sohlh1 signalling pathways. Among them, CDC42 regulates the activation of primordial follicles by regulating the expression level of PTEN in oocytes, which in turn regulates PI3K signalling activity in oocytes

The ovarian reserve is the reserve of dormant primordial follicles. The size of the follicle reserve is determined by a balance of follicle activation, survival and loss.^11^ Within this chain of events, the correct activation and genesis of the primordial follicle is a key process in the reproductive phase.^12^ In female vertebrates, multiple signalling pathways exist to control primordial follicle activation, such as the PI3K‐AKT‐mTOR signalling pathway, in which PTEN (a phosphatase and tensin homologue missing on chromosome 10) is a negative regulator on this signalling pathway, and the phosphorylation of ribosomal protein S6 (rpS6), 4EBP and IKK**α** involved downstream of mTOR levels^13^, ^14^ are also synergistically involved in primordial follicle activation. In addition, p27Kip1‐CDK signalling pathway, MDM2/p53 signalling pathway and Wee1, Nobox, Sohlh1 and other signalling pathways^15^, ^16^, ^17^, ^18^, ^19^ are also involved in the regulation of folliculogenesis. These findings can enable us to better understand the regulatory role of CDC42 in folliculogenesis and further clarify the mechanisms involved in the cascade regulation (Figure 2).

During Drosophila oogenesis, deletion of DAck (a mammalian homologue of the activated CDC42‐related kinase) results in defects in the structure and morphology of the Drosophila egg chamber, as well as impaired follicular activation, leading to reduced fertility.^20^ In the early stages of Drosophila oogenesis, increasing or decreasing CDC42 has no detrimental effect on intra‐embryonic egg chamber formation, but in later stages, it can lead to defects in cytoplasmic transfer, and thus can affect egg formation.^21^, ^22^ During primordial follicle activation in mouse ovaries, CDC42 expression increased and regulated the activation process of primordial follicles by binding to phosphatidyl alcohol‐4,5‐bisphosphate 3‐kinase catalytic subunit β (p110β) to downregulate PTEN expression levels in oocytes, which in turn regulated PI3K signalling activity in oocytes. Meanwhile, short‐term in vitro treatment experiments also showed that high CDC42 expression by transfection with lentiviral constructs significantly promoted the activation of primordial follicles in both neonatal and adult mice ovaries.^23^ Recent studies have found that in mice treated with EGF in vivo and in vitro, the growth factor EGF improves the activation of primordial follicles by increasing CDC42‐PI3K signalling activity.^24^ During female folliculogenesis, CDC42 promotes proper follicle activation by acting as a helper in the regulation of the cascade.

## 4 MEIOSIS IN OOCYTES

**FIGURE 3:**
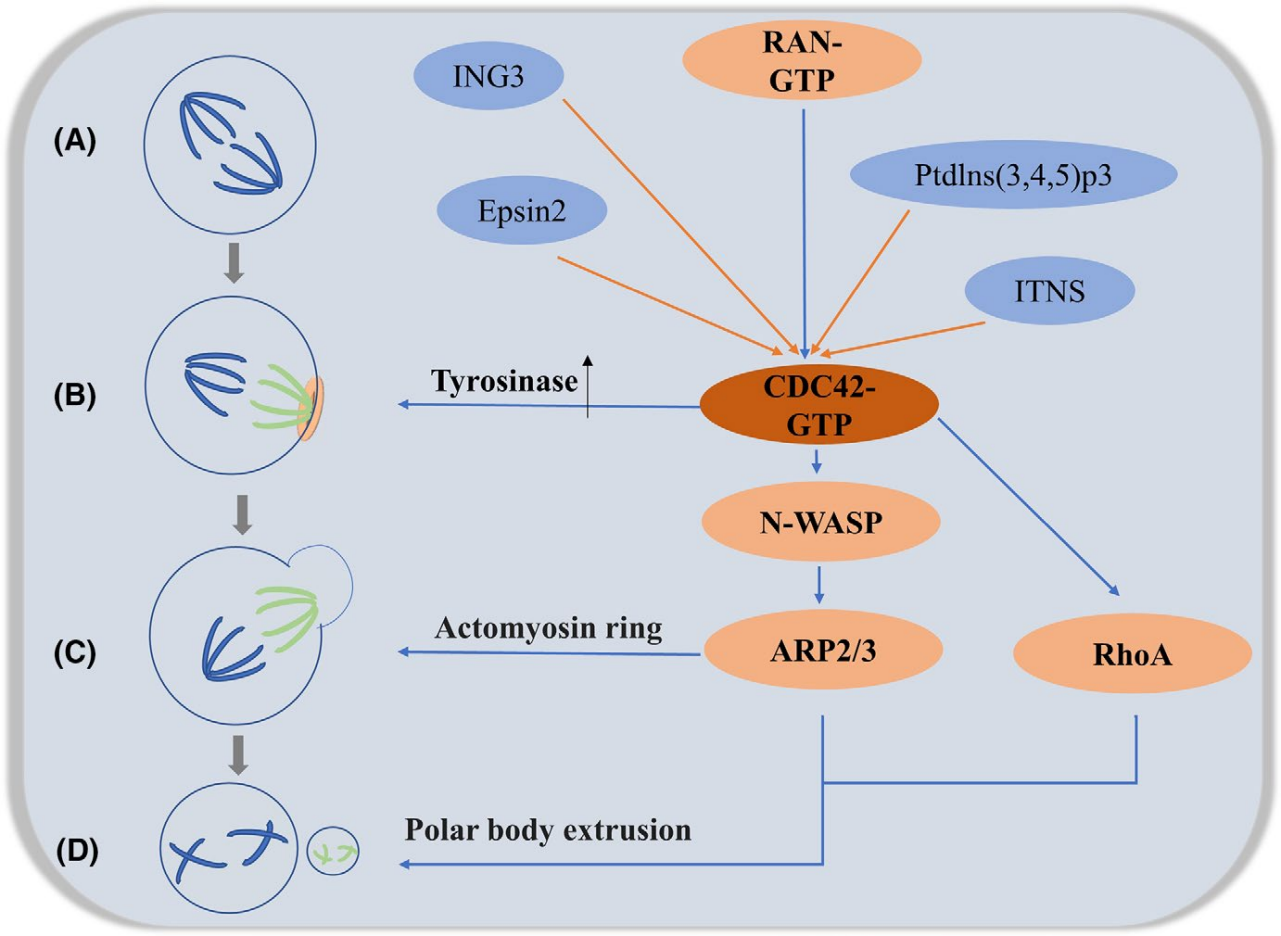
(A) The meiotic spindle is initially symmetrical and located in the centre of the oocyte. (B) The chromosomes emit a RAN‐GTP gradient and induce CDC42 signalling to form the polarized cortex (light pink). CDC42 signalling from the polarized cortex induces tyrosinylation of α‐microtubulin (light green microtubules) on the side of the spindle closest to the cortex. Microtubules emanating from the spindle pole towards the centre of the oocyte remain predominantly detyrosylated (dark blue microtubules). (C) Asymmetric positioning of the meiotic spindle is accompanied by a series of polarization events that culminate in the formation of the spindle actin cap. cdc42 can regulate its formation via the N‐WASP–Arp2/3 signalling pathway. (D) The active region of CDC42 defines the inner boundary of the RhoA loop and is propelled by actin filaments generated by the N‐WASP–Arp2/3 pathway to provide the necessary polar body efflux. At this point, the oocyte divides into two daughter cells: the secondary oocyte (after the first meiosis) or oocyte (after the second meiosis) and the polar body. During meiosis, multiple genes such as ING3, Epsin2, Ptdlns(3,4,5)p3, ITSN2 and others are involved in coregulation

Oocyte meiosis is an extreme example of cytoplasmic division, during which a single cell divides into two daughter cells, which requires extensive spatiotemporal synchronization driven by upstream regulators to ensure accuracy. In this regard, chromosome segregation must be coordinated with plasma membrane entry to produce two distinct daughter cells.^25^ Rho‐GTPase plays a major regulatory role in many signal transduction processes due to its modifiable ‘deactivation‐activation’ nature, which is mediated by covalent lipid modification (prenylation) for membrane fixation and activation. Lipid modifications bind to the plasma membrane, and GTP hydrolysis confers binary molecular ‘switching’ activity on Rho family proteins. When bound to GTP, they are active and can interact with downstream effectors; however, when bound to GDP, they are inactive.^26^ Thus, many regulatory factors control the localization activity of Rho‐GTPase by localizing themselves to different subcellular structural domains, which in turn are regulated by or are in association with the transport of microtubules and the actin cytoskeleton. As such, the spatiotemporal regulation of Rho‐GTPase and its regulators led to local modulation of downstream effectors and targeted changes in actin dynamics. The role of CDC42 in the meiotic process is summarized in Figure 3.

### 4.1 CDC42 and spindle asymmetry

The drive of meiosis consists of three conditions: asymmetry in cell fate, the functional differences between homologous chromosomes affecting their segregation and asymmetry within the meiotic spindle.^27^, ^28^ The meiotic spindle in oocytes is different from mitotic spindles in that it lacks microtubule‐organizing centres called centrosomes. Instead, meiotic chromosomes organize the microtubules of the meiosis I (MI) spindle, which forms first in the centre of the oocyte but then moves in an actin‐dependent manner perpendicularly towards the oocyte cortex.^29^ In 2017, Akera et al. found in their examination of post‐translational modifications of spindle microtubules in mid‐meiosis that tyrosine‐microtubule proteins were more abundant on the side of the cortex, suggesting that this unequal distribution may contribute to driving meiosis; furthermore, spindle bodies aligned parallel to the cortex showed more α‐microtubulin tyrosinase on the side close to the cortex region (excluding the spindle poles),^7^ suggesting that signals from the cortex directly regulate tyrosinase rather than being transmitted through the spindle poles.

In mouse germinal vesicle (GV) oocytes, CDC42 is located only in the cortex; after germinal vesicle breakdown (GVBD), it aggregates at chromosome condensation and eventually localizes to the microtubules of the meiotic spindle.^30^ The use of CDC42T17N (a negative CDC42 mutant) on mature mouse oocytes results in MI spindle elongation. These elongated spindles do not efficiently separate homologous chromosomes,^31^ and reduce tyrosine‐microtubule signalling proteins overall, thereby preventing spindle asymmetry. Active CDC42 increases α‐microtubulin tyrosinase, resulting in increased expression of tyrosine‐microtubulin.^7^ In optogenetic experiments, the targeting of active CDC42 to one pole of the symmetric meiotic spindle induced an asymmetric increase in α‐microtubulin tyrosinase, even without cortical proximity.^7^ This result suggests that the asymmetric localization of activated CDC42 relative to the spindle contributes to the asymmetric mechanism of the spindle.

The CDC42 signalling is established by a chromatin‐based gradient of RAN, a small GTPase with well‐established roles in microtubule dynamics, in addition to the cortical polarization it induces, is critical for the formation of spindle asymmetry.^27^ The eccentric intracellular localization of chromosomes during oocyte maturation induces differentiation of the overlying cortex, allowing for a structurally homogeneous reorganization of the cortical cytoskeleton adjacent to chromatin in the early meiotic state, all of which suggests that chromatin is necessary and sufficient for determining the site of division.^32^ In this process, RAN signalling helps to activate CDC42 and polarize the oocyte cortex. Abrogation of this chromosome‐directed RAN signalling is sufficient to eliminate the spindle asymmetry and disrupt biased chromosome orientation required for centromere drive in spite of the proximity of the spindle to the oocyte cortex.^7^

### 4.2 CDC42 and the formation of actin caps

During meiosis, the asymmetric positioning of the meiotic spindle is accompanied by a series of polarization events, culminating in the differentiation of the polarized zone around the spindle. Thus, a hallmark feature of oocyte polarization is the formation of the ‘actin cap,’ a thick layer of actin filaments that accumulate on the cortex of the spindle.^33^

Neural Wiskott–Aldrich syndrome protein (N‐WASP) plays an important role in cytokinesis by mediating and coordinating spindle and actin activity.^34^ Oocyte‐specific deletion of N‐WASP does not affect oocyte polarity, but it does lead to failure of the second meiotic completion. In African Xenopus oocytes, the role between N‐WASP and the Arp2/3 complex links CDC42‐dependent signalling to actin assembly. In this regard, N‐WASP interacts extensively with CDC42 and the C segment of N‐WASP binds to the Arp2/3 complex to interconnect it,^35^ and in this pathway, although N‐WASP is a weak stimulator of the Arp2/3 complex, its activity can be enhanced by upstream factors such as CDC42 and PI(4,5)p2^36^ and that CDC42 can stimulate membrane protrusion in oocytes through the ARP2/3 complex..^37^ This is in contrast to the downregulation of the Arp2/3 complex by CDC42, which inhibits oocyte membrane protrusion.^38^ In mouse oocytes, the chromatin signal Ran GTPase drives the formation of polarized actin caps by promoting the accumulation of polarized N‐WASP signalling across the spindle cortex,^39^ and the silent expression of CDC42 resulting from its diminished signalling also allows for the disruption of actin cap formation.^40^ As such, we hypothesize that the signalling cascade of oocyte actin polarization: Chromatin—Ran‐GTPase—CDC42‐GTP—N‐WASP—Arp2/3—actin cap.^41^ In this process, CDC42 is a determinant of N‐WASP localization in the mouse oocyte.^39^

The regulation of meiosis is an extremely complex regulatory network that requires the combined regulation of numerous genes. In mouse oocytes, ING3, Epsin2 and Ptdlns(3,4,5)p3 all regulate CDC42 activity, coordinate polarity establishment and cytoplasmic division in mouse oocytes and the migration of actin and spindle to the cortex, thereby regulating asymmetric cell division during mouse oocyte maturation.^42^, ^43^, ^44^; In porcine oocytes, intersectins (ITSNs) regulate the assembly of the meiotic apparatus and actin polymerization by interacting with Cdc42, thus ensuring orderly meiosis during the maturation of porcine oocytes^45^; IQGAP1 and PAK1 are involved in cytoplasmic division and regulation of chromatin condensation, respectively, as downstream mediators of CDC42^30^, ^46^, ^47^; and the mammalian target of rapamycin (mTOR), which is essential for mammalian cell proliferation, survival and viability, also inhibits spindle migration and asymmetric division during mouse oocyte maturation via the CDC42 signalling pathway.^48^ The co‐regulation of multiple genes maximizes the temporal and spatial coherence of this process and makes it much more tolerant, providing for the correct production of gametes.

### 4.3 CDC42 with Polar body emission

Animal cells use a contractile ring associated with the plasma membrane to create a division groove that divides the cell into two daughter cells.^49^ The contractile ring is a network of actin and myosin filaments, and the motor activity of myosin drives the contraction of myosin filaments by transferring actin. In mitotic cells, the movement of the contractile ring is directed by Rho‐GTPase.^50^ The inhibition of its activity using Clostridium botulinum C3 transferase, which is a specific Rho GTPase inhibitor, blocks polar body emission in females. CDC42 is the first activation contact site at the beginning of cytoplasmic division, and the active region of CDC42 defines the inner boundary of the RhoA active loop, which forms the cytokinetic contractile loop.^31^ This in turn leads to polar body extrusion.^32^, ^51^ In African Xenopus oocytes, the inhibition of Rho‐GTPase activity disrupts the distribution of microtubules and actin filaments in the spindle, reduces the rate of first polar body extrusion^5^, ^52^ and prevents polar body emission due to the inappropriate formation of the contractile ring.^31^ In mouse oocytes, Rho‐GTPase contributes to membrane protrusion during polar body formation and ring contraction.^38^ During meiosis in mouse oocytes, the Ran GTPase‐CDC42 GTP‐N WASP‐Arp2/3 signalling pathway is conserved during late meiosis, and CDC42 is an essential component of the pathway that is required for first polar body emission in vivo. In contrast, the inhibition of CDC42 signalling can lead to the release of N‐WASP into the cytoplasm, allowing secondary complete failure of second polar body extrusion in oocytes.^53^, ^54^ We speculate that actin filaments generated through the polarized CDC42/N‐WASP pathway may provide the necessary impetus (which is similar to the actin‐driven extension of the leading edge of motor cells) to deform the cortex and drive polar body protrusion.^55^, ^56^ Zhang et al. have recently also shown that CDC42 is an indispensable protein during meiosis in porcine oocytes, and that it facilitates the polar body extrusion process through actin formation.^57^

It follows that polar body emission requires RhoA contractile loops and CDC42‐mediated membrane protrusion. Active RhoA is localized in an ECT‐2‐dependent manner,^58^ prelocated on the cap of the spindle and post‐located on the contractile ring.^59^ Activation of CDC42 in the local cortex requires asymmetric spindle pole attachment and late initiation, which in turn defines the surface of the inhibitory pole body. The CDC42 active region overlaps with dynamic actin and is bounded by the RhoA contractile ring. During cytoplasmic division, contraction of the RhoA contractile ring is accompanied by CDC42‐mediated membrane movement, allowing a spindle pole and a set of chromosomes to be pulled into the oocyte cortex on the side surrounded by CDC42.^51^ At this point, the oocyte divides into two daughter cells: the secondary oocyte (after the first meiotic division) or oocyte (after the second meiotic division) and the polar body. Indeed, oocyte journey from MI to Meiosis II (MII) stage is coordinated by several factors and pathways that enable oocyte to extrude PBI. Quality of oocyte directly impacts fertilization rate, early embryonic development and reproductive outcome in mammals.^60^

## 5 CDC42 AND FERTILIZATION

Mammalian fertilization is associated with the initiation of the MII process of the oocyte, followed by the development of fertilization–gamete fusion to produce a new organism, and is the culmination of a large number of complexly regulated cellular processes. The process is associated forward with the precise regulation of oocyte recruitment and development to the ovulation of the dominant follicle, and backward with the expulsion of the oocyte dipole and protoplast fusion, and the rapid transition from the relative quiescence of oogenesis to the rapid oogenesis stage of early embryogenesis. Given the complexity of the process, the changes in CDC42 during fertilization are discussed separately for the two parts of the fertilization process that focus on cytoskeletal remodelling: sperm capacitation, acrosome reaction and MII (described in Part II).

Cytoskeletal remodelling is necessary for sperm capacitation and acrosome response. During energization, F‐actin is located in the acrosome and equatorial regions, but during the acrosomal response of spermatozoa from bulls, rats, mice and guinea pigs, F‐actin is relocalized in the post‐acrosomal region. Actin polymerization and relocalization are generally regulated by small GTPases that activate Wasp proteins, which coordinate with Arp2/3, profilin I and profilin II to accomplish cytoskeletal remodelling. Among them, small GTPases play an important role in cytoskeletal remodelling during these processes in sperm.^61^ Specifically, in mammalian sperm, the acrosome response (AR) is considered a regulated secretory event that is an essential requirement for physiological fertilization. It is an exocrine response regulated by Ca2+ that allows for a variety of membrane fusion events.^62^ During the acrosome reaction, CDC42 plays a central role in the regulated exocytosis by activating the polymerization of SNARE proteins and actin. In addition, the lipid raft protein caveolin‐1 (CAV1) functions as a scaffold for CDC42 and as a guanine nucleotide dissociation inhibitory protein, while it is inactivated when CDC42 binds to CAV1, and the activation of CDC42 facilitates the disruption of CAV1‐CDC42 interactions that are involved in regulating energetic acquisition and the acrosome response.^63^ Further studies in guinea pig sperm showed that the inhibition of CDC42 and RhoA altered the kinetics of actin polymerization, energetic acquisition and acrosome response in different ways, and that the initiation of actin polymerization and activation of RhoA were dependent on the activation of CDC42.^64^ Similarly, in the fertility phenotype of male mice with aryl hydrocarbon receptor Ahr‐knockout (Ahr‐KO), half of the few sperm produced were found to exhibit morphological alterations, while those with typical morphology may have pathological changes. Further studies showed that the production of oligo‐weak teratogenic spermatozoa was associated with low abundance of CDC42 and very limited actin polymerization during capacitation.^65^ This shows that CDC42 is the basis for normal sperm development and acrosome reaction.

## 6 CDC42 AND EARLY EMBRYONIC DEVELOPMENT

The conserved polarity effector proteins PAR‐3, PAR‐6, CDC‐42 and atypical protein kinase C (aPKC) constitute a core unit of the segmentation‐deficient (PAR) protein network and play a central role in polarizing many types of animal cells,^66^ And its study in the early nematode embryo is useful for understanding the mechanism of embryonic division in postnematode cells. In single‐cell embryos of Cryptobacterium hidradi, polarity is traditionally defined along the anterior–posterior axis by the separation of PAR proteins into anterior (PAR‐3, PAR‐6) and posterior (PAR‐1, PAR‐2) cortical domains.^67^ Whereas PAR polarization is established under the control of Rho1 and maintained by CDC42,^68^ in this process CDC42 acts in concert with PAR proteins to control polarity in nematode embryos,^69^, ^70^, ^71^ further studies have shown that in addition to its primary role in regulating the size of the precortical region through binding to PAR‐6, CDC42 plays a major role in regulating PAR‐independent 6‐dependent cortical myosin,^72^ and this evidence also provides evidence for the interaction of CDC42 with the PAR‐3/PAR‐6 complex as an evolutionarily conserved functional unit. During mouse pre‐implantation development, cell polarization first occurs within the eight‐cell stage blastocyst as part of a process called compaction. Activated CDC42 protein is involved in the polarization of early mouse oocytes by forming additional cytoplasmic actin bundles between the nucleus and cell–cell contact points during this process within the eight‐cell stage blastocyst of the mouse embryo, inducing nuclear translocation.^73^ In contrast, during late mouse embryonic development, mice with conditional knockout of the CDC42 gene in endothelial cells have an impact on embryogenesis, and researchers have found embryonic death at the stage of E7.5‐E16.5 with smaller morphology and less vascularity compared to normal embryos, this suggests that CDC42 plays an important role in embryonic development, microvascular development and microvascular permeability.^74^ On day 10, the presence of PAR3, CDC42 and aPKC was observed in the apical epithelium and lateral membranes of the porcine maternal endometrium, and on days 13 and 16, pregnancy‐induced redistribution of aPKC to the cytoplasm and redistribution of CDC42 to the apical surface of the tubule epithelium were observed, and these changes in protein distribution may limit the implantation of porcine embryos into the stroma.^75^ CDC42 is involved in the compatible implantation of embryos into the endometrial stroma from early embryonic polarity generation to later embryonic compatibility with the endometrial stroma and has an important role in the entire embryonic stage from budding to implantation.

## 7 CDC42 AND GRANULOSA CELLS

**FIGURE 4:**
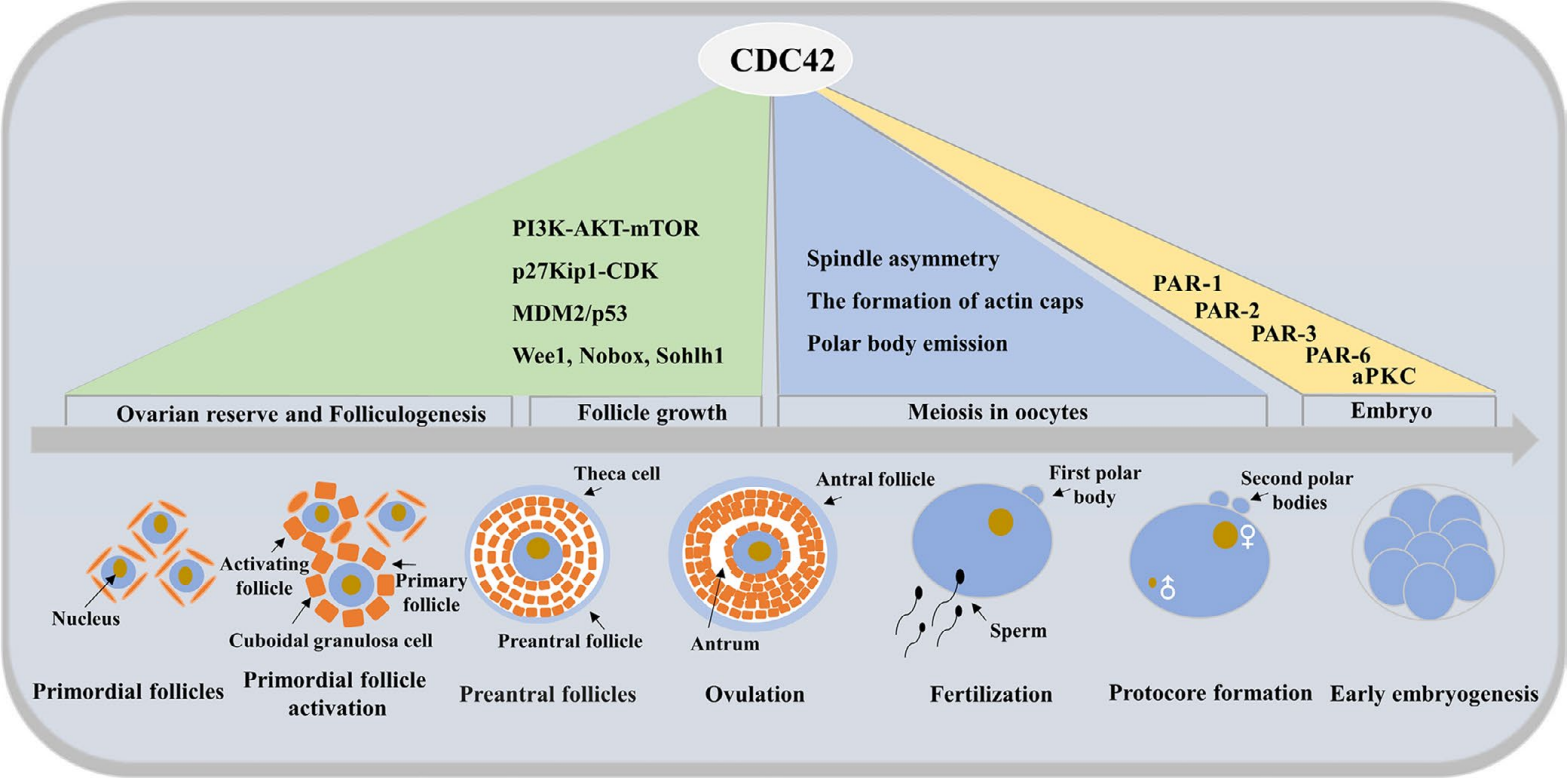
Full model map of CDC42 in the control of follicle growth. CDC42 is involved in female vertebrate gametogenesis through the combined regulation of multiple genes. Involved in ovarian reserve and follicle activation, oocyte meiosis (this part includes three parts: spindle asymmetry, actin cap formation and polar body emission), fertilization, early embryonic development and granulosa cell regulation of the microenvironment

We should not overlook the great role played by the granulosa cells, the intimate partners of the oocyte, in the regulation of female reproduction. Granulosa cells play an important role in the development and maturation of the oocyte, both in vitro and in vivo, by secreting endosteroids and growth factors that interact with the oocyte to create a highly precise microenvironment to guarantee correct gametogenesis.^76^, ^77^ In early studies, CDC42 was found to regulate the germinating activity of luteinizing hormone granulosa cells (LGCs) following human chorionic gonadotropin (hCG) stimulation and may play a role in the formation of the corpus luteum.^78^ Therefore, any alteration in granulosa cell metabolism and apoptosis may have an impact on oocyte and embryo quality.^79^ In porcine primary granulosa cells cultured in vitro, the expression of CDC42 can be upregulated to inhibit granulosa cell apoptosis.^80^ In the follicles of the chicken ovary, the expression of CDC42 can be upregulated to promote granulosa cell proliferation.^81^ In human ovarian granulosa tumour cells, miR‐23a induced cell cycle arrest via the CDC42/PAK1 pathway.^82^ Clinical trials have also observed that increasing CDC42 expression in granulosa cells from patients with polycystic ovary syndrome improves fertility.^83^ Further studies have shown that granulosa cell CDC42 expression is significantly associated with pregnancy outcome and is a potential follicle marker associated with embryos.^79^ Also of interest to us is the significant difference in CDC42 expression observed between gestation‐positive and negative transfer embryos during in vitro development.^84^, ^85^ This suggests that CDC42 not only regulates granulosa cell proliferation and apoptosis but also regulates the entire embryonic development process through the regulation of granulosa cells. Overall, CDC42 plays a key role in the total process of female gametogenesis and has a very bright performance in synergizing gametogenesis and creating a good microenvironment for oocyte growth. Its complete pattern is shown in Figure 4.

## 8 CONCLUSIONS AND PERSPECTIVES

This paper analyses and summarizes the roles and mechanisms played by CDC42 in the female vertebrate reproductive system, and clarifies the central role of CDC42 in female vertebrate gamete production. As a member of the evolutionarily conserved Rho‐GTPase family, the function of CDC42 is involved in the generation of mammalian female gametes from unicellular yeast to multicellular, and the mechanisms involved in CDC42 range from single gene activation to determine cortical localization to cascade regulation of multiple genes to trigger pole body expulsion. Similarly, CDC42 plays a dominant role in oocyte maturation by regulating the proliferation and apoptotic processes of granulosa cells. Could we use it as a biomarker to assess oocyte quality or further increase preclinical exploration with the aim of providing new drug targets for in vitro oocyte development and maturation process in the future with the aim of improving oocyte quality. From basic to clinical, there is still a long way to explore.

## CONFLICT OF INTEREST

The authors declare that the research was conducted in the absence of any commercial or financial relationships that could be construed as a potential conflict of interest.

## AUTHOR CONTRIBUTIONS

**Qiaojuan Mei:** Conceptualization (lead); Data curation (lead); Formal analysis (lead). **huiying LI:** Methodology (equal). **Yu Liu:** Methodology (equal). **Xiaofei Wang:** Methodology (equal). **wenpei Xiang:** Funding acquisition (equal); Project administration (equal).
